# Aqua­dicrotonato(di-2-pyridyl­amine)cobalt(II)

**DOI:** 10.1107/S1600536808008118

**Published:** 2008-03-29

**Authors:** Jian Wu

**Affiliations:** a College of Chemistry and Ecological Engineering, Guangxi University for Nationalities, Nanning 530006, GuangXi, People’s Republic of China

## Abstract

The Co atom in the title complex, [Co(CH_3_CHCHCOO)_2_(C_10_H_9_N_3_)(H_2_O)], has a distorted recta­ngular–pyramidal geometry formed by the chelating dipyridylamine ligand, and two O atoms of monodentate carboxyl­ate groups of two different crotonate anions and a water molecule. The complex forms a three-dimensional supra­molecular network *via* inter­molecular O—H⋯O, N—H⋯O and C—H⋯O hydrogen-bonding contacts.

## Related literature

For related literature, see: Addison *et al.* (1984 ▶); Chang *et al.* (1999 ▶); Peng *et al.* (2000 ▶); Wu (2007 ▶); Xu *et al.* (2004 ▶); Zhang (2007 ▶).
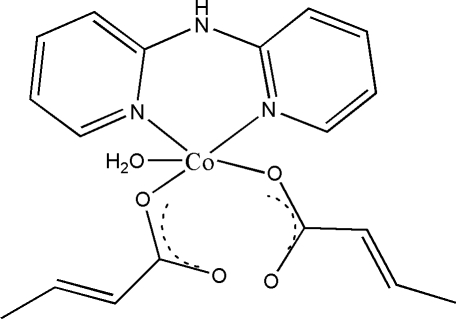


## Experimental

### 

#### Crystal data

[Co(C_4_H_5_O_2_)_2_(C_10_H_9_N_3_)(H_2_O)]
*M* *_r_* = 418.31
Monoclinic, 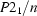

*a* = 7.1113 (7) Å
*b* = 16.8303 (15) Å
*c* = 15.9850 (14) Å
β = 91.291 (2)°
*V* = 1912.7 (3) Å^3^
*Z* = 4
Mo *K*α radiation
μ = 0.93 mm^−1^
*T* = 298 (2) K
0.28 × 0.22 × 0.19 mm

#### Data collection

Bruker APEXII area-detector diffractometer
Absorption correction: multi-scan (*SADABS*; Sheldrick, 2004 ▶) *T*_min_ = 0.781, *T*_max_ = 0.843
9697 measured reflections
3448 independent reflections
2714 reflections with *I* > 2σ(*I*)
*R*_int_ = 0.064

#### Refinement

*R*[*F* ^2^ > 2σ(*F* ^2^)] = 0.041
*wR*(*F* ^2^) = 0.107
*S* = 0.96
3448 reflections
252 parameters
2 restraints
H atoms treated by a mixture of independent and constrained refinement
Δρ_max_ = 0.34 e Å^−3^
Δρ_min_ = −0.36 e Å^−3^

### 

Data collection: *APEX2* (Bruker, 2004 ▶); cell refinement: *SAINT* (Bruker, 2004 ▶); data reduction: *SAINT*; program(s) used to solve structure: *SHELXS97* (Sheldrick, 2008 ▶); program(s) used to refine structure: *SHELXL97* (Sheldrick, 2008 ▶); molecular graphics: *SHELXTL* (Sheldrick, 2008 ▶); software used to prepare material for publication: *SHELXTL*.

## Supplementary Material

Crystal structure: contains datablocks I. DOI: 10.1107/S1600536808008118/si2078sup1.cif

Structure factors: contains datablocks I. DOI: 10.1107/S1600536808008118/si2078Isup2.hkl

Additional supplementary materials:  crystallographic information; 3D view; checkCIF report

## Figures and Tables

**Table 1:** Hydrogen-bond geometry (Å, °)

*D*—H⋯*A*	*D*—H	H⋯*A*	*D*⋯*A*	*D*—H⋯*A*
O5—H5*A*⋯O4	0.892 (15)	1.73 (3)	2.577 (4)	156 (6)
O5—H5*B*⋯O2^i^	0.888 (15)	1.86 (2)	2.729 (3)	166 (5)
N21—H21⋯O2^ii^	0.86	1.95	2.798 (3)	168
C8—H8⋯O4^iii^	0.93	2.47	3.356 (4)	160

Symmetry codes: (i) 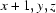; (ii) 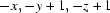; (iii) 
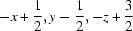
.

**Fig. 1.**
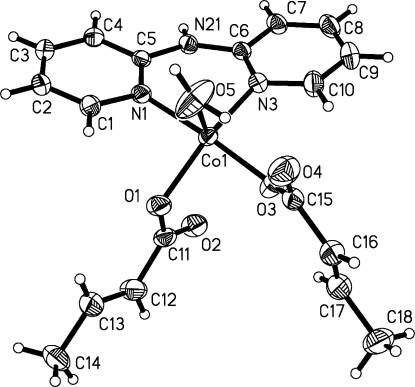
The molecular structure of the title complex, showing 30% probability displacement ellipsoids and the atomic numbering scheme. H atoms are shown as spheres of arbitrary radii.

**Fig. 2.**
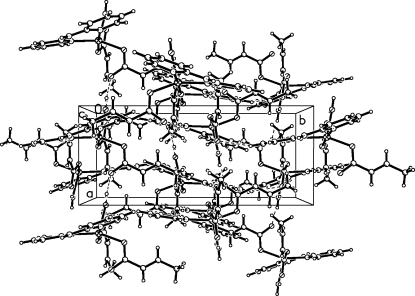
A view of the title complex, showing O—H···O and C—H···O hydrogen bonds that contribute to the construction of a three-dimensional network, with hydrogen bonds shown as dashed lines.

## supplementary crystallographic information

### Comment

Transition metal complexes with polypyridylamine ligands, possessing diverse structures and special optical and electromagnetic properties (Peng *et al.*, 2000), have aroused great interest among researchers. The pyridyldiamine ligand usually exhibits donor as well as acceptor properties and can be used as a popular chelating ligand (Chang *et al.*, 1999; Xu *et al.*, 2004).

As shown in the Scheme and Fig. 1, the Co atom in the title complex has a contorted rectangular pyramidal coordination geometry formed by the chelating dipyridine-2-ylamine (tpdaH2) ligand and two oxygen atoms of monodenate carboxylate groups of two different crotonic acid anions. The tpdaH2 ligand and the crotonic acid ligand consist of the basal plane. The coordinated water molecule hold the vertex location. The O1–Co1–N3 and O3–Co1–N1 angles are α = 156.63 (9)° and β = 175.67 (10)°, respectively. These angles were used to calculate a parameter τ, which is defined as τ = (β - α)/60 (Addison *et al.*, 1984). In the case of a perfectly tetragonal symmetry, this value is equal to zero, and for a perfectly trigonal symmetry it is 1.0. In the presented structure this value is 0.317, indicating that the polyhedron is about 70% rectangular pyramidal. The dihedral angle between the pyridine ring planes is 12.74 (8)°, which is much larger than that of our reported similar organic ligand (6.10 (15)°) (Wu, 2007). The average bond lengths with Co–N is 2.01 Å, and the Co–O bond lengths range from 1.943 (2) to 2.215 (3) Å. The bond lengths with Co–N are shorter than those of a nickel complex with 2,3'-dipyridylamine (Zhang, 2007).

In the title complex the H atoms of two NH groups of tpdaH2 act as donors to form intermolecular classical hydrogen bonds with O2 as acceptor atoms. Synchronously, the coordinated water molecule takes as donor and binds to the uncoordinated oxygen atom O2 of one of the carboxylate groups, and to the intramolecular acceptor atom O4. A weak intermolecular C—H···O contact completes the three-dimensional supramolecular network (Table 1 and Fig. 2).

### Experimental

CoSO_4_(0.022 g, 0.011 mmol), *L*(0.035 g, 0.023 mmol), tpdaH2 (0.028 mg, 0.013 mmol) and NaOH(0.048 mmol,0.12 mmol), were added in a mixed solvent of benzene and methanol, the mixture was heated for six hours under reflux. During the process stirring and influx were required. The resultant was then filtered to give a pure solution which was infiltrated by diethyl ether freely in a closed vessel. Two weeks later some single crystals of the size suitable for X-ray diffraction analysis were obtained.

### Refinement

All H atoms (except the water H atoms) were placed in calculated positions [C*sp*^2^—H and N—H = 0.93 Å and 0.86 Å, respectively, and C*sp*^3^—H = 0.96 Å] and they were refined using a riding model, with *U*_iso_(H) = 1.2*U*_eq_(C,*N*) and 1.5*U*_eq_ for the CH_3_ groups. The methyl H atoms were allowed to rotate (AFIX 137) to optimal positions. The water H atoms were found in a difference electron density map, they were refined using distance restraints (O—H = 0.900(0.015) Å), with *U*_iso_(H) = 1.5*U*_eq_(O).

### Crystal data

**Table:**

[Co(C_4_H_5_O_2_)_2_(C_10_H_9_N_3_)(H_2_O)]	*F*_000_ = 868
*M**_r_* = 418.31	*D*_x_ = 1.453 Mg m^−^^3^
Monoclinic, *P*2_1_/*n*	Mo *K*α radiation λ = 0.71073 Å
Hall symbol: -P 2yn	Cell parameters from 3448 reflections
*a* = 7.1113 (7) Å	θ = 1.8–25.2º
*b* = 16.8303 (15) Å	µ = 0.93 mm^−^^1^
*c* = 15.9850 (14) Å	*T* = 298 (2) K
β = 91.291 (2)º	Block, green
*V* = 1912.7 (3) Å^3^	0.28 × 0.22 × 0.19 mm
*Z* = 4	

### Data collection

**Table:**

Bruker APEXII area-detector diffractometer	3448 independent reflections
Radiation source: fine-focus sealed tube	2714 reflections with *I* > 2σ(*I*)
Monochromator: graphite	*R*_int_ = 0.064
*T* = 298(2) K	θ_max_ = 25.2º
φ and ω scan	θ_min_ = 1.8º
Absorption correction: multi-scan(SADABS; Sheldrick, 2004)	*h* = −7→8
*T*_min_ = 0.781, *T*_max_ = 0.843	*k* = −20→20
9697 measured reflections	*l* = −17→19

### Refinement

**Table:**

Refinement on *F*^2^	Secondary atom site location: difference Fourier map
Least-squares matrix: full	Hydrogen site location: inferred from neighbouring sites
*R*[*F*^2^ > 2σ(*F*^2^)] = 0.041	H atoms treated by a mixture of independent and constrained refinement
*wR*(*F*^2^) = 0.107	*w* = 1/[σ^2^(*F*_o_^2^) + (0.0594*P*)^2^] where *P* = (*F*_o_^2^ + 2*F*_c_^2^)/3
*S* = 0.96	(Δ/σ)_max_ = 0.001
3448 reflections	Δρ_max_ = 0.34 e Å^−^^3^
252 parameters	Δρ_min_ = −0.36 e Å^−^^3^
2 restraints	Extinction correction: none
Primary atom site location: structure-invariant direct methods	

### Special details

**Table:**

Geometry. All e.s.d.'s (except the e.s.d. in the dihedral angle between two l.s. planes) are estimated using the full covariance matrix. The cell e.s.d.'s are taken into account individually in the estimation of e.s.d.'s in distances, angles and torsion angles; correlations between e.s.d.'s in cell parameters are only used when they are defined by crystal symmetry. An approximate (isotropic) treatment of cell e.s.d.'s is used for estimating e.s.d.'s involving l.s. planes.
Refinement. Refinement of *F*^2^ against ALL reflections. The weighted *R*-factor *wR* and goodness of fit *S* are based on *F*^2^, conventional *R*-factors *R* are based on *F*, with *F* set to zero for negative *F*^2^. The threshold expression of *F*^2^ > σ(*F*^2^) is used only for calculating *R*-factors(gt) *etc*. and is not relevant to the choice of reflections for refinement. *R*-factors based on *F*^2^ are statistically about twice as large as those based on *F*, and *R*- factors based on ALL data will be even larger.

### Fractional atomic coordinates and isotropic or equivalent isotropic displacement parameters (Å^2^)

**Table:**

	*x*	*y*	*z*	*U*_iso_*/*U*_eq_	
Co1	0.19268 (5)	0.572818 (19)	0.66967 (2)	0.04151 (15)	
O1	0.0960 (3)	0.68420 (11)	0.66984 (13)	0.0643 (6)	
O2	−0.1802 (3)	0.62901 (12)	0.64366 (13)	0.0706 (6)	
O3	0.0968 (4)	0.56293 (13)	0.78204 (14)	0.0757 (7)	
O4	0.3464 (4)	0.59240 (16)	0.86323 (16)	0.0932 (8)	
O5	0.4820 (4)	0.6001 (3)	0.71532 (17)	0.1196 (12)	
H5B	0.600 (3)	0.605 (3)	0.699 (3)	0.179*	
H5A	0.468 (9)	0.598 (3)	0.7706 (11)	0.179*	
N1	0.2756 (3)	0.58881 (12)	0.55228 (14)	0.0464 (5)	
N21	0.2027 (3)	0.45990 (12)	0.50412 (13)	0.0478 (5)	
H21	0.1778	0.4334	0.4593	0.057*	
N3	0.1831 (3)	0.45414 (14)	0.65122 (14)	0.0491 (6)	
C1	0.3429 (4)	0.66182 (15)	0.53387 (18)	0.0544 (7)	
H1	0.3567	0.6983	0.5773	0.065*	
C2	0.3914 (4)	0.68510 (16)	0.45638 (19)	0.0584 (8)	
H2	0.4376	0.7359	0.4468	0.070*	
C3	0.3700 (4)	0.63086 (17)	0.39168 (19)	0.0594 (8)	
H3	0.3986	0.6455	0.3373	0.071*	
C4	0.3073 (4)	0.55626 (16)	0.40755 (17)	0.0521 (7)	
H4	0.2943	0.5192	0.3647	0.062*	
C5	0.2626 (4)	0.53625 (15)	0.48998 (16)	0.0422 (6)	
C6	0.1751 (4)	0.41803 (14)	0.57678 (18)	0.0453 (6)	
C7	0.1415 (4)	0.33680 (15)	0.5682 (2)	0.0560 (7)	
H7	0.1321	0.3141	0.5152	0.067*	
C8	0.1226 (5)	0.29138 (18)	0.6368 (2)	0.0701 (9)	
H8	0.0990	0.2372	0.6319	0.084*	
C9	0.1388 (5)	0.32670 (19)	0.7145 (2)	0.0775 (10)	
H9	0.1307	0.2965	0.7630	0.093*	
C10	0.1669 (5)	0.40655 (19)	0.7188 (2)	0.0683 (9)	
H10	0.1754	0.4299	0.7716	0.082*	
C11	−0.0798 (5)	0.68817 (17)	0.65826 (18)	0.0563 (7)	
C12	−0.1694 (5)	0.7671 (2)	0.6603 (2)	0.0754 (10)	
H12	−0.2997	0.7683	0.6644	0.090*	
C13	−0.0862 (5)	0.83305 (18)	0.6571 (2)	0.0721 (9)	
H13	0.0444	0.8315	0.6554	0.087*	
C14	−0.1757 (7)	0.91429 (18)	0.6557 (3)	0.0959 (13)	
H14A	−0.1277	0.9450	0.7021	0.144*	
H14B	−0.3096	0.9090	0.6598	0.144*	
H14C	−0.1468	0.9407	0.6043	0.144*	
C15	0.1754 (6)	0.58252 (16)	0.8511 (2)	0.0631 (9)	
C16	0.0514 (6)	0.59332 (19)	0.9235 (2)	0.0745 (10)	
H16	0.1090	0.6001	0.9758	0.089*	
C17	−0.1279 (6)	0.59403 (19)	0.9198 (2)	0.0773 (10)	
H17	−0.1859	0.5872	0.8676	0.093*	
C18	−0.2546 (7)	0.6052 (2)	0.9948 (3)	0.1070 (14)	
H18A	−0.3409	0.6480	0.9837	0.160*	
H18B	−0.1788	0.6175	1.0435	0.160*	
H18C	−0.3237	0.5572	1.0043	0.160*	

### Atomic displacement parameters (Å^2^)

**Table:**

	*U*^11^	*U*^22^	*U*^33^	*U*^12^	*U*^13^	*U*^23^
Co1	0.0445 (2)	0.0376 (2)	0.0428 (2)	−0.00619 (15)	0.00895 (16)	−0.00716 (14)
O1	0.0614 (14)	0.0476 (11)	0.0846 (15)	−0.0100 (10)	0.0218 (11)	−0.0174 (10)
O2	0.0703 (15)	0.0618 (13)	0.0802 (15)	−0.0159 (12)	0.0099 (12)	−0.0233 (11)
O3	0.0850 (17)	0.0835 (16)	0.0595 (14)	−0.0191 (13)	0.0220 (12)	−0.0124 (11)
O4	0.093 (2)	0.118 (2)	0.0690 (16)	0.0104 (17)	0.0052 (15)	−0.0033 (14)
O5	0.0630 (17)	0.228 (3)	0.0674 (17)	−0.041 (2)	0.0048 (15)	−0.017 (2)
N1	0.0458 (13)	0.0382 (12)	0.0554 (14)	−0.0025 (10)	0.0067 (11)	−0.0051 (9)
N21	0.0585 (14)	0.0348 (11)	0.0503 (13)	−0.0029 (10)	0.0036 (11)	−0.0050 (10)
N3	0.0517 (14)	0.0442 (12)	0.0516 (13)	0.0028 (10)	0.0041 (11)	0.0049 (10)
C1	0.0578 (18)	0.0402 (15)	0.0656 (19)	−0.0085 (13)	0.0111 (15)	−0.0056 (13)
C2	0.0615 (19)	0.0414 (15)	0.073 (2)	−0.0018 (14)	0.0145 (16)	0.0057 (14)
C3	0.064 (2)	0.0547 (18)	0.0603 (18)	0.0048 (15)	0.0145 (15)	0.0132 (14)
C4	0.0575 (18)	0.0479 (16)	0.0511 (16)	0.0027 (13)	0.0071 (14)	−0.0032 (12)
C5	0.0385 (14)	0.0350 (13)	0.0532 (15)	0.0028 (11)	0.0041 (12)	−0.0015 (12)
C6	0.0395 (15)	0.0384 (14)	0.0580 (17)	0.0002 (11)	0.0040 (12)	0.0024 (12)
C7	0.0597 (19)	0.0378 (15)	0.0705 (19)	−0.0017 (13)	0.0034 (15)	−0.0011 (13)
C8	0.070 (2)	0.0445 (17)	0.096 (3)	0.0004 (16)	0.0031 (19)	0.0151 (17)
C9	0.095 (3)	0.060 (2)	0.078 (2)	−0.0021 (19)	0.006 (2)	0.0249 (18)
C10	0.089 (3)	0.0603 (19)	0.0561 (19)	−0.0026 (17)	0.0034 (17)	0.0115 (15)
C11	0.061 (2)	0.0526 (17)	0.0557 (17)	−0.0032 (15)	0.0156 (15)	−0.0144 (14)
C12	0.067 (2)	0.064 (2)	0.096 (3)	−0.0001 (18)	0.0172 (19)	−0.0176 (18)
C13	0.085 (3)	0.059 (2)	0.072 (2)	−0.0010 (18)	0.0074 (18)	−0.0033 (16)
C14	0.133 (4)	0.057 (2)	0.098 (3)	0.024 (2)	0.007 (3)	0.0036 (18)
C15	0.092 (3)	0.0445 (17)	0.054 (2)	0.0105 (17)	0.0142 (19)	0.0013 (13)
C16	0.100 (3)	0.064 (2)	0.060 (2)	0.008 (2)	0.011 (2)	0.0003 (15)
C17	0.096 (3)	0.057 (2)	0.080 (2)	−0.005 (2)	0.017 (2)	−0.0024 (16)
C18	0.124 (4)	0.090 (3)	0.109 (3)	−0.006 (3)	0.056 (3)	−0.014 (2)

### Geometric parameters (Å, °)

**Table:**

Co1—O3	1.943 (2)	C4—H4	0.9300
Co1—O1	1.997 (2)	C6—C7	1.394 (3)
Co1—N1	1.998 (2)	C7—C8	1.346 (4)
Co1—N3	2.020 (2)	C7—H7	0.9300
Co1—O5	2.215 (3)	C8—C9	1.379 (5)
O1—C11	1.261 (4)	C8—H8	0.9300
O2—C11	1.244 (3)	C9—C10	1.360 (4)
O3—C15	1.269 (4)	C9—H9	0.9300
O4—C15	1.238 (5)	C10—H10	0.9300
O5—H5B	0.888 (15)	C11—C12	1.474 (4)
O5—H5A	0.892 (15)	C12—C13	1.260 (4)
N1—C5	1.334 (3)	C12—H12	0.9300
N1—C1	1.354 (3)	C13—C14	1.508 (4)
N21—C5	1.374 (3)	C13—H13	0.9300
N21—C6	1.376 (3)	C14—H14A	0.9600
N21—H21	0.8600	C14—H14B	0.9600
N3—C6	1.336 (3)	C14—H14C	0.9600
N3—C10	1.352 (4)	C15—C16	1.483 (5)
C1—C2	1.351 (4)	C16—C17	1.275 (5)
C1—H1	0.9300	C16—H16	0.9300
C2—C3	1.385 (4)	C17—C18	1.527 (5)
C2—H2	0.9300	C17—H17	0.9300
C3—C4	1.358 (4)	C18—H18A	0.9600
C3—H3	0.9300	C18—H18B	0.9600
C4—C5	1.403 (4)	C18—H18C	0.9600
			
O3—Co1—O1	87.19 (9)	C8—C7—C6	119.8 (3)
O3—Co1—N1	175.67 (10)	C8—C7—H7	120.1
O1—Co1—N1	89.07 (8)	C6—C7—H7	120.1
O3—Co1—N3	92.23 (9)	C7—C8—C9	118.8 (3)
O1—Co1—N3	156.63 (9)	C7—C8—H8	120.6
N1—Co1—N3	90.34 (8)	C9—C8—H8	120.6
O3—Co1—O5	93.20 (10)	C10—C9—C8	118.7 (3)
O1—Co1—O5	97.04 (13)	C10—C9—H9	120.6
N1—Co1—O5	89.44 (10)	C8—C9—H9	120.6
N3—Co1—O5	106.31 (13)	N3—C10—C9	124.0 (3)
C11—O1—Co1	112.95 (18)	N3—C10—H10	118.0
C15—O3—Co1	128.7 (2)	C9—C10—H10	118.0
Co1—O5—H5B	143 (4)	O2—C11—O1	123.2 (3)
Co1—O5—H5A	101 (4)	O2—C11—C12	118.6 (3)
H5B—O5—H5A	115 (5)	O1—C11—C12	118.2 (3)
C5—N1—C1	117.3 (2)	C13—C12—C11	126.1 (4)
C5—N1—Co1	126.52 (17)	C13—C12—H12	116.9
C1—N1—Co1	116.10 (18)	C11—C12—H12	116.9
C5—N21—C6	131.9 (2)	C12—C13—C14	126.9 (4)
C5—N21—H21	114.0	C12—C13—H13	116.5
C6—N21—H21	114.0	C14—C13—H13	116.5
C6—N3—C10	116.1 (3)	C13—C14—H14A	109.5
C6—N3—Co1	125.48 (18)	C13—C14—H14B	109.5
C10—N3—Co1	118.2 (2)	H14A—C14—H14B	109.5
C2—C1—N1	124.1 (3)	C13—C14—H14C	109.5
C2—C1—H1	117.9	H14A—C14—H14C	109.5
N1—C1—H1	117.9	H14B—C14—H14C	109.5
C1—C2—C3	117.9 (3)	O4—C15—O3	125.7 (3)
C1—C2—H2	121.1	O4—C15—C16	117.4 (3)
C3—C2—H2	121.1	O3—C15—C16	116.9 (4)
C4—C3—C2	120.0 (3)	C17—C16—C15	125.2 (4)
C4—C3—H3	120.0	C17—C16—H16	117.4
C2—C3—H3	120.0	C15—C16—H16	117.4
C3—C4—C5	118.7 (3)	C16—C17—C18	124.9 (4)
C3—C4—H4	120.7	C16—C17—H17	117.6
C5—C4—H4	120.7	C18—C17—H17	117.6
N1—C5—N21	120.9 (2)	C17—C18—H18A	109.5
N1—C5—C4	121.9 (2)	C17—C18—H18B	109.5
N21—C5—C4	117.2 (2)	H18A—C18—H18B	109.5
N3—C6—N21	121.0 (2)	C17—C18—H18C	109.5
N3—C6—C7	122.5 (3)	H18A—C18—H18C	109.5
N21—C6—C7	116.5 (3)	H18B—C18—H18C	109.5
			
O3—Co1—O1—C11	−76.9 (2)	Co1—N1—C5—C4	173.3 (2)
N1—Co1—O1—C11	100.9 (2)	C6—N21—C5—N1	−11.6 (4)
N3—Co1—O1—C11	12.2 (3)	C6—N21—C5—C4	169.0 (3)
O5—Co1—O1—C11	−169.8 (2)	C3—C4—C5—N1	1.7 (4)
O1—Co1—O3—C15	−84.9 (3)	C3—C4—C5—N21	−178.9 (3)
N3—Co1—O3—C15	118.5 (3)	C10—N3—C6—N21	−175.6 (3)
O5—Co1—O3—C15	12.0 (3)	Co1—N3—C6—N21	10.4 (4)
O1—Co1—N1—C5	−140.0 (2)	C10—N3—C6—C7	3.5 (4)
N3—Co1—N1—C5	16.6 (2)	Co1—N3—C6—C7	−170.5 (2)
O5—Co1—N1—C5	123.0 (3)	C5—N21—C6—N3	9.1 (4)
O1—Co1—N1—C1	36.6 (2)	C5—N21—C6—C7	−170.0 (3)
N3—Co1—N1—C1	−166.8 (2)	N3—C6—C7—C8	−2.5 (5)
O5—Co1—N1—C1	−60.5 (2)	N21—C6—C7—C8	176.7 (3)
O3—Co1—N3—C6	157.8 (2)	C6—C7—C8—C9	−0.6 (5)
O1—Co1—N3—C6	69.7 (3)	C7—C8—C9—C10	2.2 (5)
N1—Co1—N3—C6	−18.8 (2)	C6—N3—C10—C9	−1.8 (5)
O5—Co1—N3—C6	−108.3 (2)	Co1—N3—C10—C9	172.7 (3)
O3—Co1—N3—C10	−16.1 (3)	C8—C9—C10—N3	−1.1 (6)
O1—Co1—N3—C10	−104.2 (3)	Co1—O1—C11—O2	−3.8 (4)
N1—Co1—N3—C10	167.4 (2)	Co1—O1—C11—C12	177.7 (2)
O5—Co1—N3—C10	77.8 (3)	O2—C11—C12—C13	−164.5 (3)
C5—N1—C1—C2	2.3 (4)	O1—C11—C12—C13	14.1 (5)
Co1—N1—C1—C2	−174.6 (2)	C11—C12—C13—C14	177.4 (3)
N1—C1—C2—C3	0.3 (5)	Co1—O3—C15—O4	−20.5 (5)
C1—C2—C3—C4	−1.9 (5)	Co1—O3—C15—C16	160.5 (2)
C2—C3—C4—C5	0.9 (4)	O4—C15—C16—C17	171.8 (3)
C1—N1—C5—N21	177.4 (2)	O3—C15—C16—C17	−9.0 (5)
Co1—N1—C5—N21	−6.1 (4)	C15—C16—C17—C18	−180.0 (3)
C1—N1—C5—C4	−3.2 (4)		

### Hydrogen-bond geometry (Å, °)

**Table:**

*D*—H···*A*	*D*—H	H···*A*	*D*···*A*	*D*—H···*A*
O5—H5A···O4	0.892 (15)	1.73 (3)	2.577 (4)	156 (6)
O5—H5B···O2^i^	0.888 (15)	1.86 (2)	2.729 (3)	166 (5)
N21—H21···O2^ii^	0.86	1.95	2.798 (3)	168
C8—H8···O4^iii^	0.93	2.47	3.356 (4)	160

Symmetry codes: (i) *x*+1, *y*, *z*; (ii) −*x*, −*y*+1, −*z*+1; (iii) −*x*+1/2, *y*−1/2, −*z*+3/2.
